# Zinc Application Mitigates Copper Toxicity by Regulating Cu Uptake, Activity of Antioxidant Enzymes, and Improving Physiological Characteristics in Summer Squash

**DOI:** 10.3390/antiox11091688

**Published:** 2022-08-29

**Authors:** Farhad Behtash, Fatemeh Abedini, Hosein Ahmadi, Seyed Bahman Mosavi, Ahmad Aghaee, Mohammad Reza Morshedloo, Jose M. Lorenzo

**Affiliations:** 1 Department of Horticultural Science, Faculty of Agriculture, University of Maragheh, Maragheh 83111-55181, Iran; 2 Department of Horticulture Science, College of Agriculture and Natural Resources, University of Tehran, Karaj 77871, Iran; 3 Department of Soil Science and Engineering, Faculty of Agriculture, University of Maragheh, Maragheh 83111-55181, Iran; 4 Department of Biology, Faculty of Science, University of Maragheh, Maragheh 83111-55181, Iran; 5 Centro Tecnológico de la Carne de Galicia, Rúa Galicia Nº 4, Parque Tecnológico de Galicia, San Cibrao das Viñas, 32900 Ourense, Spain; 6 Área de Tecnoloxía dos Alimentos, Facultade de Ciencias, Universidade de Vigo, 32004 Ourense, Spain

**Keywords:** summer squash, copper toxicity, zinc application, antioxidant enzymes

## Abstract

Zinc (Zn) and copper (Cu) are essential micronutrients for the plant’s growth, development, and metabolism, but in high concentrations, the elements disrupt normal metabolic processes. The present study investigated the effects of different concentrations (added to a Hogland-based solution) of zinc (control, 5, 10 mg L^−1^ ZnSO_4_) and copper (control, 0.1, 0.2 mg L^−1^ CuSO_4_) on the growth characteristics and biochemical indices of summer squash (*Cucurbita pepo* L.). Compared with control, a single application of Cu or Zn at both concentrations significantly declined fruit yield, growth traits, pigments content, and high content of these minerals and values of stress-related indices. Increased Cu concentration in the nutritional solutions reduced the activity of ascorbate peroxidase (APX) and guaiacol peroxidase (GPX). Copper at high concentrations intensified ROS production, aggravated oxidative stresses, and decreased the plant yield and productivity. Nonetheless, combining Cu and Zn could alleviate stress intensity by boosting antioxidant enzymes, redox regulation, and a resultant diminishment in the content of H_2_O_2_, proline, malondialdehyde, and minerals. The obtained results corroborate that the co-application of zinc in Cu-contaminated areas can improve the plant’s economic yield and physiological parameters by hindering copper toxicity and enhancing the photosynthetic capacity.

## 1. Introduction

As expected, by 2050, the world population will exceed 10 billion; thus, meeting the food requirements of this enormous population will be one of the future hub challenges for administrations [1]. The agriculture sectors in most parts of the world suffer from inclement climate and environmental stresses (i.e., biotic and abiotic kinds), deepened by the worldwide changes in meteorological conditions [2,3,4]. Abiotic stresses such as high temperatures, cold stress, water deficiency, salinity, and heavy metals extensively affect crop yield and productivity in arable farms [5]. The widespread anthropogenic activities such as consumption of excessive chemical fertilizers, fungicides, herbicides, and industrial emissions have been also consistently one of the leading causes of enhancement of heavy metals pollution within industrial and agricultural zones [6]. Essential heavy metals such as Mn, Fe, Co, Ni, Cu, Zn, and Mo are urgent micronutrients needed for growth, stress tolerance, photosynthesis, and biosynthesis of different biomolecules in plants; however, these elements are biologically toxic above threshold concentrations [7]. Decontamination of polluted ecosystems has become a significant concern for environmental activists since these elements can conjugate in food chains and threaten the lives of numerous species [7]. Plant reactions and metabolism changes in response to HM exposition depend on the kind of contaminant element, plant species, stress intensity, and their duration [8]. Heavy metal stresses generally leave oxidative bursts, obstruct photosynthetic carbon reduction (PCR), hinder electrons transport chain, and thereby stop ATP production in plant cells [9]. In HM-polluted soils, the microbiome variation is noticeably changed; thus, this event can substantially influence the plant growth and interactions with these microorganisms [9].

Copper (Cu) is an essential trace element, biologically found in the structure of plastocyanin proteins, and is required for activating various kinds of essential enzymes such as oxidase (e.g., cytochrome oxidase ӀV, ascorbic acid oxidase, and polyphenol oxidase) and superoxide dismutase [10]. Under physiological conditions, a copper ion can exist in oxidized, cupric (Cu^2−^) or reduced, cuprous (Cu^−^) form. Plants grown in the presence of a high concentration of Cu (more than 30 mg kg^−1^ dry weight) show decreased yield and chlorosis symptoms [6]. Free Cu can provoke oxidative shock and intervene in critical cellular processes such as photosynthesis, chlorophyll biosynthesis, rubisco enzyme activity, the effectiveness of PSII, membrane stability, and other metabolic pathways [11]. Furthermore, Cu-induced perturbation in the stomatal function adversely influences photosynthetic machinery and results in abridged carbon dioxide intake [12]. Both cupric and cuprous ions can greatly actuate oxidation and reduction reactions [13]. It has been proposed that in the presence of superoxide radicals or reducing compounds such as ascorbic acid or glutathione, the cupric ion can be reduced to a cuprous form, which is responsible for the production of hydroxyl radicals from hydrogen peroxide (H_2_O_2_) through the Weiss and Haber reaction [13]. The hydroxyl radicals are the most potent destructing free radicals in biological systems and can degenerate every biological molecule [14]. Under oxidative attack, the plant promotes its radical scavenging system against inorganic contaminants with the help of antioxidants enzymes such as superoxide dismutase (SOD), catalase (CAT), and ascorbate peroxidase (APX) to rejuvenate the developing parts of plants [15,16,17].

Zinc (Zn), another necessary micronutrient for plant growth and development, participates in various well-known biochemical processes such as the fixing of ribosomal fractions, transcription of DNA through zinc-based metalloproteins, activating fundamental enzymes involved in photosynthesis and CO_2_ sequestration, and the biosynthesis of auxin, chlorophylls, and proteins [18]. The deficiency of zinc elements in the world’s agricultural soils, especially in calcareous surfaces, is an ordinary matter of global concern, highlighting the importance of fortifying poor soils with zinc-based fertilizers [19]. Zn deficiency usually occurs in high pH soils rich in calcium carbonates and salts and under over-consumption of phosphate fertilizers [19]. Adding ZnSO_4_ may amend this condition with further acidification of anaerobic soils [20]. Different micro-nutrients interact with zinc (Zn) by influencing each other’s uptake, bioavailability, and quantitative status in plants. Zn has a potential antagonism with copper and manganese, and these interactions may increase or decrease plant growth and development [21]. By taking advantage of this interplay between nutritional elements, farmers can confront with HMs toxicity and soil contamination [20].

*Cucurbita pepo* L., commonly known as summer squash, is an annual herbaceous plant with many health-promoting effects [22]. The production value of the plant passed 28 million tonnes in 2020, strengthening the immediate need for studying this species (Food and Agriculture Organization, 2020) [23]. Therefore, the present study intended to examine the impact of Zn supplementation on the activity of antioxidant enzymes, elements concentration, and status of growth parameters in summer squash under copper stress conditions.

## 2. Materials and Methods

### 2.1. Experimental Design and Treatments

To evaluate the impacts of different concentrations of Cu (0, 0.1, and 0.2 mg L^−1^ CuSO_4_·5H_2_O) and Zn (0, 5, and 10 mg L^−1^ ZnSO_4_·7H_2_O) on morpho-physiological characteristics of summer squash, a factorial experiment based on randomized complete block design (RCBD) with triplicate was carried out in a greenhouse located in the faculty of agriculture, University of Maragheh, Iran. The seeds of *C. pepo* were purchased from Nickerson-Zwaan Company, France. Seeds were decontaminated in 3% (*v*/*v*) sodium hypochlorite for about three min and finally washed with distilled water. Squash seeds were sown in 12 L pots containing coco-peat: perlite (1:1 *v*/*v*) as an inert media. Plants were water-irrigated until reaching the 4-leaves stage. Then, the established plants were fed daily with nutrient solutions. For this purpose, different amounts of CuSO_4_ and ZnSO_4_ salts were weighted, and then these salts were added to a modified Hogland-based nutrient solution (Table 1). For the control treatment, only a basal nutrient solution was used for irrigation. Finally, enough amounts of leaf samples were frozen in liquid nitrogen and kept at −80 °C until biochemical analysis.

### 2.2. Growth Characteristics and Fruit Yield

To obtain dry weights of fruit and leaves, plant Samples were oven-dried at 70 °C for two days and then weighted. For measurement of leaf area, fully expanded middle leaves were separated, and the leaf area was determined using a commercial leaf area meter (Delta-T, Cambridge, UK).

### 2.3. Antioxidant Enzymes Activity

Kinetic assessment of Catalase (CAT), Guaiacol peroxidase (GPX), and ascorbate peroxidase (APX) enzymes activity were carried out using the outlines and details described by related studies [2,15,25]. The enzyme activity was expressed as Unit g^−1^ FW min^−1^.

### 2.4. Proline, MDA, and H_2_O_2_ Content

The proline content of fresh leaves was estimated using a ninhydrin reagent [26]. Following the procedure, total proline content was dissolved in the toluene phase and then prepared for spectrophotometric measurements in a 96-well quartz plate. Finally, the spectrums of samples were recorded by a plate reader at 520 nm. Total proline content was expressed as μmol proline g^˗1^ fresh weight.

Malondialdehyde (MDA) content as an indicator of oxidative reaction intensity in biological membranes was determined following the method described by Heath and Packer [27]. According to provided protocol, thiobarbituric acid (TBA) reagent was used to produce MDA-TBA adducts, which show the maximum absorbance at 532 nm and 600 nm. After measuring the absorbance of samples, MDA content was determined using its extinction coefficient of 155 mM cm^−1^ and expressed as nmol g^−1^ fresh weight (FW).

Fresh leaves’ hydrogen peroxide (H_2_O_2_) content was quantified using the potassium iodide method [2]. The absorbance was read at 390 nm. Peroxide hydrogen content was determined according to the standard calibration curve and expressed as μmol g^−1^ fresh weight (FW).

### 2.5. Photosynthetic Pigments Content

The total contents of chlorophyll *a*, chlorophyll *b*, and carotenoids were estimated according to the method explained by Arnon [28]. In this procedure, 0.2 g of powdered fresh leaves was immersed in 10 mL 80% acetone; then, samples were centrifuged for 10 min at 8000 rpm. The absorption spectra of prepared samples were recorded at 663, 645, and 470 nm, and the total contents of each pigment were calculated using the following Equations. (Equations (1)–(3)):

Chlorophyll *a* content (mg g^−1^ FW) = [12.7 (A663) − 2.69 (A645)] × V/1000 W (1)

Chlorophyll *b* content (mg g^−1^ FW) = [22.9 (A645) − 4.68 (A663)] × V/1000 W (2)

Carotenoids content (mg g^−1^ FW) = [(A480) + (0.114 × A663) − (0.638 × A645)] × V/1000 W (3)

In these equations, V represents the volume of consumed acetone, and W represents the weight of fresh leaves. Furthermore, the total chlorophyll value was read by a single-photon avalanche diode (SPAD) device (502 Plus Chlorophyll Meter Japan). For this, three plants from each treatment were randomly selected, and then the value of three leaves from each plant was recorded by the device. The mean value of these measurements was reported.

### 2.6. Measurement of Cu, Zn, and Mn Content

To measure the element concentration, plant samples were initially washed with deionized water and then oven-dried at 70 °C for two days. One gram of dried fruits, roots, and leaves was ground and passed a 30-mesh screen. Then, 10 mL nitric acid (65%) was added to the samples to complete the digestion procedure. In this step, samples were exposed to 70 °C for 5 h in a thermal bath. The extracts were filtered through Whatman no. 40 papers, and the volume of the final samples was adjusted to 100 mL. An atomic absorption spectrophotometer determined the element’s content (Shimadzu, AA6300, Kyoto, Japan). Standard and sample solutions of Cu, Zn, and Mn were prepared. By optimizing spectroscopic conditions, a steady baseline was acquired. Spectrums were recorded by measuring the absorbance of each element at its related wavelength using mineral-specific lamps. After calibration, the concentration of the elements was finally reported as mg kg^−1^ dry weight [29].

### 2.7. Statistical Analysis

Analysis of variance (ANOVA) and mean comparisons (Duncan test) were performed using SAS 9.4 software (SAS Institute, Cary, NC, USA). The multivariate analysis was conducted through Xlstat version 2019 software (Addinsoft, Paris, France).

## 3. Results and Discussion

### 3.1. The Effects of Zinc and Copper on Growth Parameters

**Figure 1:**
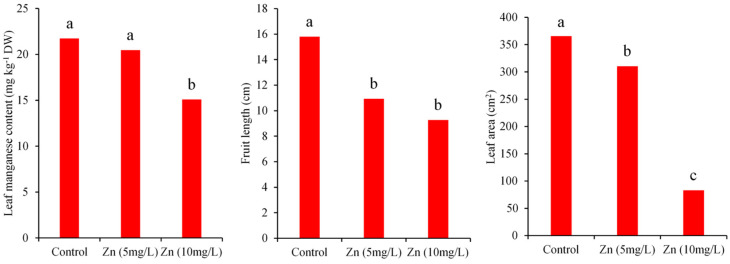
Main effects of added Zn concentration on leaf Mn content, fruit length, and leaf area of *C. pepo* L. The plot represents means of triplicates. Different alphabetic letters on each bar show significant differences at the 5% level between simple effects of treatments according to Duncan’s multiple range mean comparison test.

**Figure 2:**
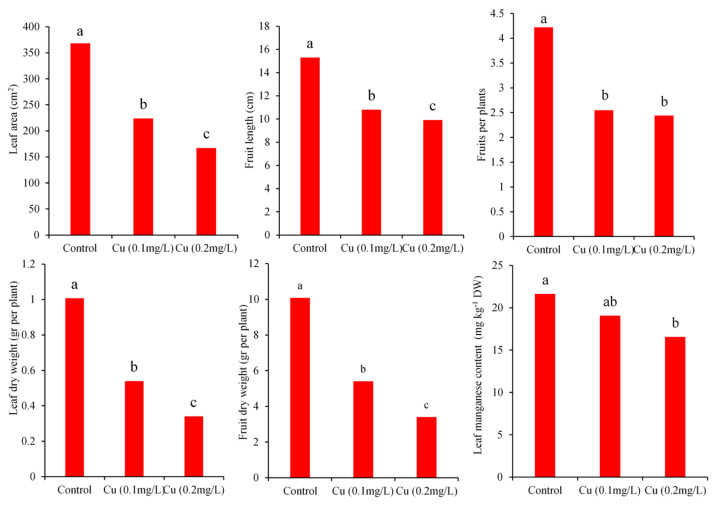
Main effects of added Cu concentration on growth traits and leaf Mn content of *C. pepo* L. The plot represents means of triplicates. Different alphabetic letters on each bar show significant differences at the 5% level between simple effects of treatments according to Duncan’s multiple range mean comparison test.

The results of variance analysis indicated that the interaction effects of leaf area and fruit length are significant at the 1% level. The simple effects of Zn and Cu on leaf dry weight, fruit length, and leaf area were statistically significant at 1%. Moreover, the simple effects of copper on dry fruit weight and fruit number were also significant (*p*-value < 0.01). As presented in Figure 1 and Figure 2, single supplementation with Cu or Zn at both concentrations significantly reduced the value of the growth traits mentioned above compared to control.

**Figure 3:**
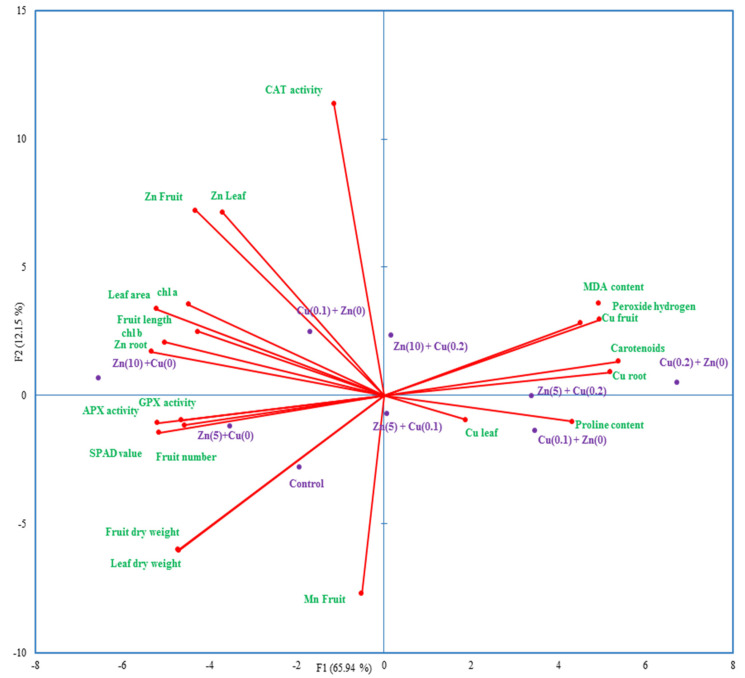
Principal component analysis (PCA) based on the correlation method unties the relationship between treatments and measured traits.

The highest values of fruit and leaf dry weight were obtained by control treatments without any addition of Cu and Zn to the nutrient solution, as confirmed by principal component analysis (Figure 2 and Figure 3). Notably, fruit dry weight was decreased by about 186%, and 296% under copper application at 0.1 and 0.2 mg L^−1^, respectively (Figure 2). These findings are well-matched with the results of the Dong et al. [29] study, which revealed that the application of copper at high concentrations could result in a diminishment of shoot dry weight, fruit number, and leaf area in tomato.

**Figure 4:**
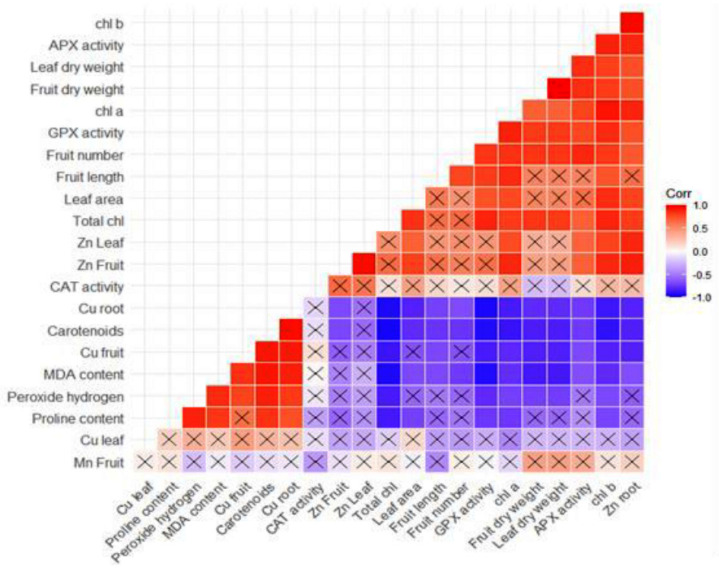
Heat-map plot showing Pearson correlation intensities between measured characteristics. The sign × stands for statistically insignificant correlations.

Meanwhile, another study demonstrated that adding nano- and bulk copper to soils at high concentrations could have an adverse effect growth characteristics of *C. pepo* [11]. The reduction in biomass production of plants under copper stress is mainly attributable to oxidative stresses, disrupted absorption of other elements, and reduced metabolism [30]. In the case of the single application of Zn or co-supplementation of Cu and Zn, leaf area and dry weight values were significantly enhanced compared with the control treatment (data are not shown). Zinc is a crucial micro-nutrient that plays a pivotal role in activating functional enzymes such as carbonic anhydrases, dehydrogenases, and DNA/RNA polymerases, as well as in the biosynthesis of auxin as growth-regulating hormones [10]. Thus, the mentioned functions of Zn are well-consistent with the finding of this study, which showed that co-application of zinc with copper could negate copper adverse effects and improve growth parameters. Furthermore, correlation analysis disclosed a positive correlation (r = 0.83, *p*-value < 0.01) between fruit number and leaf dry weight (Figure 4). This correlation means that biomass production directly affects fruit yield and productivity.

### 3.2. The Effects of Zinc and Copper on Antioxidant Enzymes Activity

ANOVA displayed that the simple effects of copper and zinc treatments on antioxidant enzymes (i.e., CAT, GPX, and APX) were significant (*p*-value < 0.01). Furthermore, the interaction effects of copper and zinc treatments on catalase and guaiacol peroxidase were also significant (*p*-value < 0.01). Overall, the mean comparison of main effects showed that copper at both concentrations upturns the activity of antioxidant enzymes. In confirmation, Azooz et al. [31] indicated that Cu supplementation at toxic levels induces ROS production in chloroplast and elevates catalase activity in wheat. APX, a key enzyme involved in the *glutathione*-ascorbate (ASC-GSH) cycle, is an H_2_O_2_-scavenging enzyme in chloroplast and cytosol [32]. In addition, the catalase enzyme is responsible for the decomposition of H_2_O_2_ stored in peroxisomes [33]. The outputs of the mean comparison analysis demonstrated that a single application of copper ascended catalase activity, whereas it reduced GPX and APX activity (Table 2). In another piece of literature, the authors declared a similar behavior in summer squash by showing a significant downturn in the activity of the APX enzyme by about 80% when exposed to nano- and bulk copper treatments [11]. In support of our findings, the report of Faizan et al. [1] demonstrated that single or blended application of zinc nanoparticles and 24-epibrassinolide in tomatoes alleviates copper toxicity by promoting antioxidant enzyme activity such as catalase and superoxide dismutase. Comparing the main effects, copper application at 0.2 mg L^−1^ enhanced the activity of CAT, GPX, and APX by about 133%, 64%, and 52%, respectively, which was considerably higher than the control. However, the co-application of copper and zinc decreased GPX and APX activity, probably due to the reduction in Cu toxicity.

Furthermore, a single application of zinc could up-regulate the activity of these three antioxidant enzymes (Table 2 and Table 5). By application of Zn at 10 mg L^−1^, CAT, GPX, and APX activity were increased by about 250%, 24%, and 92%, respectively (Table 2). Zn is one of the ribosome’s main components that support the functional structure of this protein-synthesizing machine [34]. The results mentioned above can be interpreted in this sense that the boosted activity of the antioxidant enzymes under Zn supplementation is possibly due to the increased expression of these proteins through enough support of Zn. On the other hand, zinc can regulate gene expression levels in a parallel line with protein expression by suppressing RNase activity as mRNA degrading enzymes [34].

The heatmap plot showed significant negative correlations between stress-related indices (i.e., MDA, proline, and hydrogen peroxide content and Cu concentration), GPX, and APX activity. As illustrated in Figure 3, GPX and APX activity is at a close angle with Zn(10) + Cu(0) treatment, and loading bars related to CAT activity are close to Zn(10) + Cu(0.2), showing that these enzymes are in their highest activity at these points. Despite the up-regulation of CAT and APX under Cu toxicity, any reduction in H_2_O_2_ content was not observed (Table 2). Therefore, it can be inferred that scavenging free radicals are beyond the up-limit power of these enzymes to negate over-produced ROS and peroxide hydrogen. However, the promising application of zinc, along with gradual enhancements in Cu concentration, not only regulated the activity of antioxidant enzymes but also could reduce H_2_O_2_ content and membrane peroxidation.

### 3.3. The Effects of Zinc and Copper on the Content of Photosynthetic Pigments

Except for the Cu×Zn interactive effect on chlorophyll content, all simple and interaction effects of Zn and Cu treatments had a significant influence at the 1% level on chlorophyll *a*, chlorophyll *b*, and carotenoid as well as the SPAD value as an indication of estimated total chlorophyll content. According to Table 3, values related to chlorophyll *a* and *b* content are substantially decreased along with enhancement of Cu concentration compared with their respective control. Our previous study well-concurred with the present study’s findings by showing that drought-induced oxidative damage decreases chlorophyll content [33,35]. Moreover, carotenoid content increased when plants were exposed to increased Cu concentrations (Table 3). Under copper stress predicament, carotenoids are the first-line defenders against oxidative damage to chlorophyll [35]. Carotenoids and chlorophyll *b* both play an important protective role through scavenging singlet oxygen (^1^O_2_) and anion superoxide (O^−2^), which seriously damage reaction centers in photosynthetic complexes [33]. In a study, the authors declared that copper stress induces enhanced expression of some genes involved in photosynthesis, carotenoid biosynthesis, and carbon assimilation [35]. Co-application of copper and zinc reduced SPAD value and chlorophyll *b* content, whereas carotenoid content increased (Table 3). Mean comparisons of the main effects depicted that Zn application enhanced the content of chlorophyll *a*, *b*, and total chlorophyll but decreased carotenoid content. Cu supplementation showed the reverse behavior compared with Zn. As shown in Figure 4, the SPAD value, and chlorophyll *a* and *b* content had significant negative correlations with stress-related indices (i.e., MDA, proline, H_2_O_2_, carotenoids, and Cu content), while carotenoid content indicated positive correlations with these indices. HM stress disrupts the electron transport chain and produces reactive oxygen species, which can rapidly harm biomolecules and biological membranes [36]. The long-term existence of HMs in plant cells eventually instigates a lowered level of photosynthesis, chlorophyll destruction, plant senescence, and programmed cell death [35]. HMs also impede Fe absorption, which plays a pivotal role in the biosynthesis pathway of chlorophyll and carbon assimilation [37].

### 3.4. The Effects of Zinc and Copper on the Content of Photosynthetic Pigments

Analysis of variance manifested that the simple effect of Zn and interaction effect (Cu×Zn) on MDA content was significant at the 5% level. The simple effect of Cu on MDA content was significant at the 1% level. Moreover, simple effects of Cu and Zn and their interaction effect on proline and H_2_O_2_ content were also significant at the 1% level. A single application of Cu increased the proline, H_2_O_2_, and MDA amount, whereas a single application of Zn does not significantly differ from the control (Table 2). However, the mean comparison of simple effects showed that Zn decreased the value of the indices mentioned above, while Cu behaved reversely. Co-application of these elements effectively reduced proline, H_2_O_2_, and MDA content in comparison with a single treatment of Cu (Table 2). Co-application of Zn at 10 mg L^−1^ combined with both concentrations of Cu yielded better results than 5 mg L^−1^ Zn and showed a better detoxification function (Table 2). Zn application at 10 mg L^−1^ reduced proline, H_2_O_2_, and MDA content by about 377%, 52%, and 27%, respectively (Table 5). Proline serves as a compatible solute and provides enough nitrogen resources to plant cells under environmental stresses [2]. Accumulated proline under heavy metal contamination acts as a stress reliever through ROS quenching and regulating turgor pressure [38]. H_2_O_2_, another stress-related bio-marker, has an exclusive role in several stress-resistance mechanisms such as strengthening cell walls (i.e., lignification and cross-linking between cell-related structural proteins) and phytoalexins production [39]. Hydrogen peroxide is a crucial regulator in many physiological circumstances such as senescence, photo-respiration, stomatal conductance, and cell cycle [39]. Indeed, H_2_O_2_ is known for its dual functionality, acting as a destructing molecule in high concentrations, but, in lower amounts, emits signaling signs to activate defensive mechanisms against an imminent stressful condition [2,38].

Interestingly, correlation analysis unraveled a negative relationship between antioxidant enzymes, pigments, growth characteristics, and stress-related revealers (proline, H_2_O_2_, and MDA content) (Figure 4). The negative relationships between these toxic products and the up-raised activity of antioxidant enzymes approve that zinc application may lead to cell detoxification by decomposing H_2_O_2_ and other free radicals. In reaction with sulfhydryl groups of membranes, Zn stabilizes biological membranes and can affect their permeability. The negative relationship between MDA content (indicative of peroxidation severity in bio-membranes) and Zn content in different tissue substantiates this claim.

### 3.5. The Effects of Zinc and Copper on Cu, Zn, and Mn Concentrations in Roots, Leaves, and Fruits

According to ANOVA, the simple effects of Cu and Zn application on Cu, Zn, and Mn concentrations in different tissue were significant at the 1% level. Analysis of variance depicted that the interaction effects are also significant, except for leaf Mn content. The mean comparison of simple effects showed that leaf manganese content under the application of 10 mg L^−1^ Zn (15.80 mg kg^−1^ DW) has a significantly lower value when compared with the control (21.74 mg kg^−1^ DW) (Figure 1). Referring to Figure 2, the application of Cu at the highest level showed a similar diminishment in the content of leaf manganese. Manganese plays a vital role in the gradual evolution of oxygen in PSӀӀ, fatty acids biosynthesis, nitrogen metabolism, and the function of phosphate carriers [40]. Plants under manganese deficiency may encounter reduced cell elongation and biomass yield [40]. Shahriaripour and Tajabadipour [21] showed that Zinc application severely antagonized the uptake of manganese and copper and had conflicting effects on the absorption of Fe in pistachio.

Another study confirmed our results by showing that the increased concentration of Zn can result in an intense depression of Mn in shoots [9]. The outcomes of a study on tomatoes agreed with our results by demonstrating that a higher concentration of Cu can also reduce Zn and Mn uptake [41]. Overall, the accumulation of measured elements in leaves was significantly higher than that of fruits and roots (Table 5). It can be assumed that disseminating these elements from leaves to fruit is somewhat lagging. The results demonstrate that different copper uptake mechanisms and corresponding transport pathways must operate in leaves compared with roots or fruits. Noticeably, the changing trend of mineral concentration under different treatments was more significant in root tissues when compared with leaves and fruits. Upon single utilization of each experimental factor (i.e., Cu or Zn), as much as concentrations of the applied element were elevated, an enhancement in tissue-specific contents of these elements was observed (Table 4 and Table 5).

Nonetheless, the concentration of none of these elements was not higher than the permissible values [6,23,42]. Furthermore, by a single application of copper, a partial reduction in Zn and Mn contents of different tissues was remarkably found (Table 4). Cu application at 0.2 mg L^−1^ could slightly decrease Mn content, whereas zinc application at both concentrations could insignificantly enhance Mn content in fruits (Table 4). The mean comparison of simple effects demonstrated that fruit Mn content is significantly higher than control treatment only under 5 mg L^−1^ application of ZnSO_4_ (Table 5). Moreover, co-application of zinc could markedly decrease Cu uptake and increase Zn and Mn concentrations in different tissues (Table 4). This competitive absorption can be attributed to similar binding sites assigned for Zn and Cu on the root’s surface (Mousavi et al., 2012). It was found that Zn supplementation at 10 mg L^−1^ is more effective than 5 mg L^−1^ Zn in reducing Cu absorption (Table 4). The results of the mean comparison of main effects also manifested that Zn application at 5 mg L^−1^ could not lessen leaf Cu and fruit Mn content, and their values were considerably higher than control (Table 5).

Regarding mean values of simple effects, Cu application at both concentrations significantly reduced Zn and Mn content in all three tissues (Table 5 and Figure 3). A thread of research articles proposes strong antagonism interactions among Zn, Mn, and Cu elements [6,10,11]. As shown in Figure 4, correlation analysis entangled a complicated relationship between mineral content and other measured characteristics. The Cu content of roots and fruits showed negative relationships with Zn contents of different tissues and vice versa (Figure 4). There were also significant negative correlations between Cu content and antioxidant enzymes and growth parameters (Figure 4).

## 4. Conclusions

The results of this study manifested that an increase in Zn and Mn can be better absorbed in presence of lower concentrations of CuSO_4_, whereas a Zn application at low concentration can result in a higher uptake of Cu and Mn. Moreover, the accumulation of Cu and Zn in leaves was higher than in that of fruits and roots. This incidence can be attributed to a plant defense mechanism to avoid accumulating these minerals in reproductive parts such as fruits. It was also concluded that the growth yield and dry weight of fruits and leaves are substantially influenced by the concentration of consumed Cu and Zn fertilizers. Furthermore, the elevation of the Cu concentration in basal nutrient solution left a harsh enhancement in the MDA and H_2_O_2_ content, which, in turn, up-regulated the activity of CAT to suppress free radicals and oxidative stress. Copper in high concentrations directly affects the reaction centers of photosystem ӀӀ and interrupts photosynthetic processes by annihilating photosynthetic pigments, the stability of membrane, and photosynthetic enzymes. As known, any disruption in photosynthesis efficiency compromises the final yield and economic efficiency of horticultural crops. In this regard, antagonist interaction of zinc with copper can usefully negate all destructive effects of copper toxicity. This research showed that Zn supplementation at 10 mg L^−1^ gave good results and better detoxified the noxious effects of copper than that of the 5 mg L^−1^ concentration. Thus, this study highly recommends applying Zn in Cu-contaminated soils to achieve a well-fulfilled production of summer squash.

## Figures and Tables

**Table 1:** Modified Hogland-based formulation used as a basal nutritional solution [24].

Concentration (mg L^−1^)	Micro-Element Providing Salts	Concentration (g L^−1^)	Macro-Element Providing Salts
2.86	H_3_BO_3_	0.47	Ca(NO_3_)_2_·2H_2_O
1.81	MnCl_2_·4H_2_O	0.3	KNO_3_
0.22	ZnSO_4_·7H_2_O	0.25	MgSO_4_,7H_2_O
0.02	NaMOO_4_·2H_2_O	0.06	NH_4_H_2_PO_4_
0.08	CuSO_4_·5H_2_O	0.1	Fe-EDTA

**Table 2:** Means related to the interaction effects of added Cu and Zn to a basal nutritional solution on biochemical properties of *C. pepo*.

Treatments	Proline Content (nmol g^−1^ FW)	Peroxide Hydrogen (µmol g^−1^ FW)	MDA Content (µmol g^−1^ FW)	CAT Activity (Unit g^−1^ FW min^−1^)	GPX Activity (Unit g^−1^ FW min^−1^)	APX Activity (Unit g^−1^ FW min^−1^)
**Control**	0.41 ± 0.07 c	0.33 ± 0.03 e	1.17 ± 0.13 d	0.04 ± 0.00 d	0.29 ± 0.00 a,b	1.16 ± 0.12 c
**Zn(0) + Cu(0.1)**	1.36 ± 0.07 a	0.81 ± 0.01 b	2.08 ± 0.74 ab	0.05 ± 0.02 c,d	0.17 ± 0.02 c	0.71 ± 0.13 e
**Zn(0) + Cu(0.2)**	1.52 ± 0.26 a	1.3 ± 0.17 a	2.54 ± 0.16 a	0.09 ± 0.00 b,c	0.06 ± 0.04 d	0.67 ± 0.00 e
**Zn(5) + Cu(0)**	0.32 ± 0.05 c	0.42 ± 0.14 de	1.13 ± 0.06 d	0.08 ± 0.01 c,d	0.31 ± 0.07 a	1.4 ± 0.00 b
**Zn(5) + Cu(0.1)**	0.4 ± 0.03 c	0.48 ± 0.04 d,e	1.49 ± 0.02 c,d	0.1 ± 0.01 b,c	0.19 ± 0.07 b,c	0.81 ± 0.01 de
**Zn(5) + Cu(0.2)**	0.62 ± 0.14 b	0.55 ± 0.03 c,d	1.98 ± 0.3 b,c	0.13 ± 0.00 a,b	0.16 ± 0.04 c	0.72 ± 0.03 e
**Zn(10) + Cu(0)**	0.47 ± 0.05 b,c	0.41 ± 0.03 d,e	1.2 ± 0.00 d	0.14 ± 0.01 a,b	0.36 ± 0.06 a	2.23 ± 0.23 a
**Zn(10) + Cu(0.1)**	0.39 ± 0.04 c	0.48 ± 0.02 d,e	1.85 ± 0.01 b,c	0.14 ± 0.01 a	0.21 ± 0.01 b,c	0.95 ± 0.00 d
**Zn(10) + Cu(0.2)**	0.46 ± 0.01 b,c	0.69 ± 0.05 b,c	1.54 ± 0.28 b,c,d	0.15 ± 0.00 a	0.27 ± 0.02 a,b	0.71 ± 0.00 e

In each column, means ± standard deviations having the same alphabetic letters do not have significant difference (*p* < 0.05) from each other according to Duncan’s multiple range test.

**Table 3:** The means related to interaction effects of added Cu and Zn to a basal nutritional solution on photosynthetic pigments of *C. pepo*.

Treatments	Total Chlorophyll Indicator (SPAD Value)	Chlorophyll *b* (mg g^−1^ FW)	Carotenoids Content (mg g^−1^ FW)
**Control**	41.57 ± 1.52 c,d	5.83 ± 1.30 c	4.76 ± 0.26 d,f
**Zn(0) + Cu(0.1)**	28.13 ± 6.00 e	1.41 ± 0.35 d	10.55 ± 1.46 b
**Zn(0) + Cu(0.2)**	19.44 ± 0.51 f	0.68 ± 0.34 d	15.84 ± 1.59 a
**Zn(5) + Cu(0)**	51.33 ± 2.18 a	9.2 ± 1.31 ab	3.56 ± 0.40 f,g
**Zn(5) + Cu(0.1)**	43.33 ± 2.36 b,c	5.3 ± 0.53 c	7.28 ± 1.62 c,d
**Zn(5) + Cu(0.2)**	29.49 ± 2.96 e	1.73 ± 0.40 d	10.74 ± 1.10 b
**Zn(10) + Cu(0)**	47.93 ± 4.68 a,b	12.93 ± 1.23 a	2.84 ± 0.70 g
**Zn(10) + Cu(0.1)**	39.52 ± 0.99 c,d	7.81 ± 0.90 b,c	5.71 ± 0.52 e
**Zn(10) + Cu(0.2)**	37.58 ± 3.40 d	4.92 ± 0.81 c	7.91 ± 0.54 c

In each column, means ± standard deviations having the same alphabetic letters do not have significant difference (*p* < 0.05) from each other according to Duncan’s multiple range test.

**Table 4:** Means related to the interaction effects of added Cu and Zn to a basal nutritional solution on Cu, Zn, and Mn content of *C. pepo*.

Treatments	Fruit Cu (mg kg^−1^ DW)	Leaf Cu (mg kg^−1^ DW)	Root Cu (mg kg^−1^ DW)	Fruit Zn (mg kg^−1^ DW)	Leaf Zn (mg kg^−1^ DW)	Root Zn (mg kg^−1^ DW)	Fruit Mn (mg kg^−1^ DW)
**Control**	0.77 ± 0.16 f	0.75 ± 0.04 d,e,f	0.11 ± 0.01 d	4.91 ± 0.20 e	3.43 ± 1.11 e	0.21 ± 0.01 c	5.6 ± 1.41 a,b
**Zn(0) + Cu(0.1)**	1.48 ± 0.24 d,e	1.47 ± 0.07 c,d	0.15 ± 0.01 c	4.26 ± 0.37 e	6.64 ± 2.51 d,e	0.18 ± 0.01 d	6.31 ± 0.12 a
**Zn(0) + Cu(0.2)**	4.01 ± 0.09 a	3.97 ± 0.35 a	0.29 ± 0.02 a	2.95 ± 0.73 e	7.35 ± 1.01 c,d,e	0.15 ± 0.02 e	5.19 ± 1.27 a,b
**Zn(5) + Cu(0)**	0.56 ± 0.09 e,f	0.55 ± 0.22 e,f	0.04 ± 0.02 e	16.58 ± 2.30 c	17.3 ± 1.15 b	0.27 ± 0.02 b	6.25 ± 0.30 b
**Zn(5) + Cu(0.1)**	1.26 ± 0.95 c,d	1.25 ± 0.22 d,e	0.13 ± 0.01 c,d	12.27 ± 0.32 d	11.83 ± 1.76 c	0.23 ± 0.02 c	6.08 ± 0.16 a
**Zn(5) + Cu(0.2)**	3.26 ± 0.82 b	3.28 ± 0.19 b	0.19 ± 0.02 b	5.76 ± 2.50 e	9.10 ± 1.48 c,d	0.15 ± 0.01 e	6.73 ± 0.29 a
**Zn(10) + Cu(0)**	0.21 ± 0.07 f	0.21 ± 0.11 f	0.03 ± 0.02 e	31.61 ± 2.61 a	38.8 ± 6.95 a	0.35 ± 0.02 a	6.6 ± 0.78 a
**Zn(10) + Cu(0.1)**	1.14 ± 0.06 d,e,f	1.16 ± 0.18 d,e	0.12 ± 0.03 d	27.74 ± 2.98 b	35.63 ± 3.16 a	0.28 ± 0.02 b	4.76 ± 0.57 b
**Zn(10) + Cu(0.2)**	2.01 ± 0.43 bc	2.01 ± 0.14 c	0.11 ± 0.01 d	15.77 ± 2.79 c	11.4 ± 1.51 c	0.19 ± 0.02 d	1.95 ± 0.52 c

In each column, means ± standard deviations having the same alphabetic letters do not have significant difference (*p* < 0.05) from each other according to Duncan’s multiple range test.

**Table 5:** Main effects of Cu and Zn on traits measured in the present experiment.

Main Effects of Treatments (Mean Values of Main Effects)						
Traits	Added Concentration of ZnSO_4_·7H_2_O to Basal Nutrient Solution			Added Concentration of CuSO_4_·5H_2_O to Basal Nutrient Solution		
	Control	5 mg L^−1^	10 mg L^−1^	Control	0.1 mg L^−1^	0.2 mg L^−1^
**Proline content** **(nmol g^−1^ FW)**	1.1 ± 0.54 a	0.45 ± 0.16 a	0.44 ± 0.05 b	0.4 ± 0.08 c	0.72 ± 0.49 b	0.87 ± 0.51 a
**Peroxide hydrogen** **(µmol g^−1^ FW)**	0.81 ± 0.43 a	0.48 ± 0.1 a	0.53 ± 0.13 a	0.38 ± 0.09 c	0.59 ± 0.17 b	0.85 ± 0.36 a
**MDA content** **(µmol g^−1^ FW)**	1.93 ± 0.71 a	1.53 ± 0.4 b	1.53 ± 0.31 b	1.17 ± 0.08 c	1.8 ± 0.45 a	2.02 ± 0.49 a
**CAT activity** **(Unit g^−1^ FW min^−1^)**	0.06 ± 0.02 c	0.1 ± 0.03 b	0.14 ± 0.01 a	0.08 ± 0.04 c	0.1 ± 0.04 b	0.12 ± 0.03 a
**GPX activity** **(Unit g^−1^ FW min^−1^)**	0.17 ± 0.11 b	0.22 ± 0.09 b	0.28 ± 0.07 a	0.32 ± 0.07 a	0.19 ± 0.04 b	0.16 ± 0.1 b
**APX activity** **(Unit g^−1^ FW min^−1^)**	0.85 ± 0.25 c	0.98 ± 0.32 b	1.3 ± 0.71 a	1.6 ± 0.5 a	0.83 ± 0.12 b	0.7 ± 0.03 c
**Total chlorophyll indicator (SPAD value)**	29.71 ± 10.14 b	41.38 ± 9.82 a	41.68 ± 5.59 a	46.94 ± 5.07 a	37 ± 7.59 b	28.83 ± 8.19 c
**Chlorophyll *a*** **(mg g^−1^ FW)**	15.6 ± 4.93 b	17.75 ± 2.92 b	24.15 ± 5.25 a	23.25 ± 5.4 a	18.25 ± 4.66 b	16.01 ± 4.81 b
**Chlorophyll *b*** **(mg g^−1^ FW)**	2.64 ± 2.51 c	5.41 ± 3.32 b	8.55 ± 3.62 a	9.32 ± 3.27 a	4.84 ± 2.85 b	2.45 ± 1.97 c
**Carotenoids content** **(mg g^−1^ FW)**	10.39 ± 4.92 a	7.19 ± 3.27 b	5.49 ± 2.26 c	3.72 ± 0.94 c	7.85 ± 2.42 b	11.5 ± 3.62 a
**Fruit Cu content** **(mg kg^−1^ DW)**	2.09 ± 1.48 a	1.69 ± 1.37 a	1.12 ± 0.81 b	0.51 ± 0.26 c	1.3 ± 0.52 b	3.09 ± 0.99 a
**Leaf Cu content** **(mg kg^−1^ DW)**	2.06 ± 1.47 b	3.34 ± 1.85 a	1.13 ± 0.79 c	2.15 ± 2.52 b	1.29 ± 0.2 c	3.09 ± 0.89 a
**Root Cu content** **(mg kg^−1^ DW)**	0.18 ± 0.08 a	0.12 ± 0.07 b	0.08 ± 0.04 c	0.06 ± 0.04 c	0.13 ± 0.02 b	0.19 ± 0.08 a
**Fruit Zn content** **(mg kg^−1^ DW)**	4.04 ± 0.96 c	11.54 ± 5.02 b	25.04 ± 7.55 a	17.7 ± 11.72 a	14.76 ± 10.45 b	8.16 ± 6.14 c
**Leaf Zn content** **(mg kg^−1^ DW)**	5.81 ± 2.32 c	12.74 ± 3.84 b	28.61 ± 13.5 a	19.84 ± 15.84 a	18.04 ± 13.57 a	9.28 ± 2.11 b
**Root Zn content** **(mg kg^−1^ DW)**	0.18 ± 0.03 c	0.22 ± 0.05 b	0.27 ± 0.07 a	0.28 ± 0.06 a	0.23 ± 0.04 b	0.16 ± 0.02 c
**Fruit Mn content** **(mg kg^−1^ DW)**	5.7 ± 1.07 b	6.35 ± 0.37 a	4.44 ± 2.1 b	6.15 ± 0.93 a	5.72 ± 0.79 b	4.62 ± 2.23 b

In each row, means with the same letters, either related to ZnSO_4_ or CuSO_4_ treatments do not have significant difference from each other according to Duncan’s multiple range test.

## Data Availability

The data are contained within the article.
